# Assessment of Stress and Anxiety in Parents of Neonates Admitted in a Tertiary Care Neonatal Intensive Care Unit (NICU)

**DOI:** 10.7759/cureus.66100

**Published:** 2024-08-03

**Authors:** Aayushi Joshi, Bindu Agarwal, Vasu Saini, Chaitanya Kumar Javvaji

**Affiliations:** 1 Pediatrics, Graphic Era Institute of Medical Sciences, Dehradun, IND; 2 Pediatrics, Shri Guru Ram Rai Institute of Medical and Health Sciences, Dehradun, IND; 3 Pediatrics, Jawaharlal Nehru Medical College, Datta Meghe Institute of Higher Education and Research, Wardha, IND

**Keywords:** experience in critical care, newborn, state trait anxiety inventory, neonatal intensive unit, parental stress

## Abstract

Background

A newborn’s admission into the Neonatal Intensive Care Unit (NICU) is one unexpected event capable of causing much stress and anxiety among parents. The current study aims to evaluate and compare parental stress and anxiety levels between mothers and fathers.

Methodology

This cross-sectional study was conducted in the NICU of a tertiary care center, in Uttarakhand where a total of 306 mothers and fathers were enrolled. Data was compiled using a questionnaire consisting of demographic details of parents and infants' clinical profiles, Parental Stressor Scale (PSS) NICU, and State-Trait Anxiety Inventory (STAI) scales were used to evaluate stress and anxiety, respectively.

Results

Mothers mean stress levels were greater and statistically significant. The most affected subscale in both parents was the change in parental role (M: 4.4/F: 3.3; p < 0.001). Parents with high trait anxiety also had high state anxiety (M: 51.3/F: 45.5; p < 0.001). Mothers were found to have higher trait and state anxiety than fathers.

Conclusion

Parents of newborns admitted in the NICU experience significant stress and anxiety. Mothers had higher levels of stress and anxiety; similar findings have been recorded previously in Indian and western literature.

## Introduction

Admission of a newborn into the Neonatal Intensive Care Unit (NICU) is one experience that has the potential of causing a significant amount of mental trauma, stress, and anxiety to parents as the prospects of a healthy infant are shattered by the shock of being separated from the newborn due to NICU admission [1-4]. The NICU's dense environment and an infant's sensitivity and fragility might create barriers to parenting that can have a detrimental effect on parent-infant bonding, which is the cornerstone of a lifetime relationship [5]. 

Preterm, severely unwell, and other high-risk newborns who had little hope in life are now more likely to survive because of new developments in neonatal intensive care and high-tech healthcare. More parents are exposed to the NICU setting and its range of stresses as smaller and sicker infants survive [6]. Parental stress is a well-researched and extensively debated topic in Western literature. Because of the high birth rate in India, NICU staff members are overworked and have less time to address parental concerns and psychological interventions to help reduce anxiety. Using the Parental Stressor Scale (PSS) NICU scale and the State-Trait Anxiety Inventory (STAI), the stress and anxiety levels of parents of newborns receiving NICU care were measured and compared in this study.

The aim of this study is to assess the psychological well-being of parents whose newborns are admitted to the NICU. The objectives of the research are threefold. First, it seeks to evaluate the level of stress that parents experience when their infants are admitted to the neonatal critical care unit. Second, the study aims to gauge the level of anxiety these parents feel during their newborns NICU stay. Finally, it intends to compare the levels of stress and anxiety experienced by fathers and mothers, providing a comprehensive understanding of how each parent is affected by this challenging situation.

## Materials and methods

Study design, study period, and study participants

The current cross-sectional study was carried out over 17 months, from June 2021 to October 2022, at the NICU of SMIH Hospital, a tertiary care facility in Dehradun, Uttarakhand. After obtaining informed consent, 306 mothers and fathers and 612 parents were registered. Following the newborn's admission and at least three days of exposure to the NICU setting, parents were questioned. The STAI scale, created by Spielberger in 1970, was used to examine anxiety levels, while Miles's PSS NICU scale was used to measure stress levels and gather information about the clinical profile and demographics of the infants.

Study procedure and data collection

The assessment of parental stress and anxiety was done using the PSS NICU and STAI scales, respectively. The interview of the parents of the newborn was held in a private room within the NICU. Assessment for stress and anxiety was done at least three days after NICU admission and was completed in 30-45 minutes. The collected data was recorded in a centralized sheet for analysis. The data for this study was collected under three sections. 

Section A

It included the parent's sociodemographic information (age, religion, place of residence, type of family, level of education, employment, number of children, and prior experience with a NICU hospitalization).

Section B

It consisted of the neonate's clinical profile obtained from NICU medical records (day of life; sex; mode of delivery; period of gestation; appearance, pulse, grimace, activity, and respiration (APGAR) score at birth; birth weight; the reason for hospitalization; and presence of various treatment interventions like radiant warmers, intravenous fluids, sedation, inotropes, lines, drains, ventilators, nasogastric or orogastric tubes, oxygen support, and phototherapy)

Section C

It consists of the printed PSS NICU and STAI scale questionnaire. There are 45 items in the PSS NICU scale, broken into four subscales. Every PSS NICU subscale response was graded using a five-point Likert scale. Points 1, 2, 3, 4, and 5 on the scale indicated as no stress at all, little stress, moderate stress, considerable stress, and severe stress, respectively. After calculating the overall mean score, greater mean values indicated higher stress levels. The PSS NICU scale was scored as follows: looks and behaves = 0-95; parental role = 0-50; sights and sounds = 0-25; and staff communication = 0-55. Higher scores correspond to higher levels of stress. The scores range from 0 to 225. The mean value cutoff > 2.9 was used to determine the substantial stress levels [7].

The STAI is the second scale employed in this research. The 20 statements on the STAI assist in identifying situational or present anxiety. People use the scale to indicate their feelings at any given time. The four-point scale, which goes from "not at all" to "somewhat," "moderately so," to "very much so," represents the strength of a sensation on a scale of 1 to 4, respectively. 

The 20-item STAI measure indicates a person's overall propensity to worry in anxious situations. On a four-point rating scale, participants must indicate how frequently they occur, ranging from "almost never" to "sometimes" and "often" to "almost always." Scores ranging from 20 to 80 were observed for mothers' and dads' state and trait anxiety, respectively. Higher scores represent a parent's higher level of anxiety. The mean scores were used to determine anxiety, with trait scores exceeding the 44-point cutoff and state scores exceeding the 41-point cutoff being deemed significant [6].

*Inclusion Criteria* 

Parents of newborns admitted to NICU during the study period who were hospitalized for at least three days and whose parents visited the newborn at least once during the course of their stay in NICU were eligible to participate in the study after giving written informed consent. 

Exclusion Criteria

Parents with multiple newborns (multiple births) admitted to NICU, infants with congenital anomalies, orphans and medicolegal babies, minor mothers less than 18 years of age, parents with marital discord, cognitive impairment, chronic drug addictions, psychiatric illness, and any chronic or systemic illness were excluded from the study.

Ethical consideration and statistical analysis

Ethical committee approval was obtained for the study. Parents' information was gathered through an interview using a structured questionnaire created with MS Word and MS Excel (Microsoft Corporation, Redmond, Washington, United States). A software called IBM SPSS Statistics for Windows, Version 21 (Released 2012; IBM Corp., Armonk, New York, United States) was used to perform the statistical analysis. A p-value of less than 0.5 was regarded as statistically significant.

## Results

In the study population, the average age of mothers was 25.4 years, whereas the average age of fathers was 29.7 years. The study's newborns had a mean birth weight of 2.2 kg, a gestational age of 35 weeks, and an APGAR score of 6.5 at one minute of birth. Table 1 and Table 2 summarize the details of the infant's clinical profile and demographics.

**Table 1:** Description of the demographic profile of infants

Sociodemographic variables	n (306)	n (%)
Religion		
Hindu	225	73.5%
Muslim	63	20.5%
Others	18	5.8%
Residence		
Uttarakhand	241	78.7%
Uttar pradesh	27	8.8%
Others	38	12.3%
Locality		
Urban	221	72.3%
Rural	85	27.7%
Type of family		
Joint	108	35.2%
Nuclear	198	64.8%
Education of mother		
Illiterate	0	0%
Primary	101	32.9%
Secondary/above	205	67.1%
Occupation of mother		
Professional/clerical	72	23.7%
Skilled/semi-skilled labor class	234	76.2%
Occupation of father		
Professional/clerical	146	47.7%
Skilled/semi skilled labor class	160	52.3%
Gravida		
Primigravida	62	20%
Multigravida	245	80%

**Table 2:** Description of infant clinical data PICC: Peripherally inserted central catheter; UVC: umbilical vein catheter

Infants' profile	n	n(%)
Sex		
Male	192	62.9%
Female	114	37.1%
Gestation		
Term	151	49.3%
Preterm	155	50.7%
Birth weight		
Appropriate for gestational age	220	71.7%
Small for gestational age	77	25.1%
Large for gestational age	5	1.6%
Mode of delivery		
Normal vaginal delivery	180	58.8%
Lower segment cesarean section	126	41.2%
Treatment intervention		
Radiant warmer	306	100%
Intravenous fluids	284	92.8%
Oxygen support	201	65.6%
Orogastric or nasogastric tubes	239	78.1%
PICC/UVC line	180	58.8%
Respiratory support	159	51.9%
Sedatives	67	21.8%
Catheters	58	18.9%
Phototherapy	47	15.3%
Inotropes	22	7.1%
Drains	8	2.6%
Reason for hospitalization		
Prematurity	155	50.4%
Respiratory insufficiency	119	47%
Sepsis	41	13.3%
Birth asphyxia	39	12.7%
Neonatal jaundice	38	12.3%
Shock	15	4.8%
Others	46	15%

Table 3 and Table 4 show the different PSS NICU components and the associated parental stress scores. The population's stress levels were assessed by taking the mean stress score over all four subscales, with a cutoff value of 2.9. 

**Table 3:** Comparison between the stress levels of mothers and fathers in various PSS NICU subscales PSS: Parental Stressor Scale; NICU: Neonatal Intensive Care Unit; SD: standard deviation; CI: confidence interval

PSS NICU scale	Mother mean (SD)	Father mean (SD) % with significant stress	95% CI of mean difference		p-value
	% with significant stress		Lower	Upper	
Sights and sounds	3.34 (0.899)	2.79 (0.884)	0.40678	0.68995	<0.001
	68.9%	52.2%			
Baby looks and behaves	1.68 (0.627)	1.651 (0.419)	-.05263	.11696	0.457
	2.9%	3.2%			
Parental role	4.426 (0.313)	3.332 (0.489)	1.0288	2.2696	<0.001
	100%	90.1%			
Staff behavior and communication	0.009 (0.034)	0.002 (0.014)	0.00324	0.01161	0.001
	0%	0%			
Total PSS NICU score	9.46 (1.352)	7.78 (1.187)	1.4799	1.8842	<0.001

Mothers had statistically significant stress levels with higher mean values (p < 0.001) as compared to fathers (mothers: 9.46/7.78). Maximum scores in both parents were obtained in alteration in parental role (mothers: 4.4/fathers: 3.3) where 100% of mothers had significant stress scores in contrast to 90% of fathers. It was followed by sights and sounds subscale (mothers: 3.3/fathers: 2.7) which caused significant stress in over 68.9% of mothers and 52.2% of fathers. 

The subscale most affected was parental role alteration in both the parents which was significant (p < 0.001). Maternal stress was significant in all subscales expect one (baby looks and behaves: p = 0.457) where fathers were found be to as stress as mothers (equal mean scores, mothers: 1.6). 

To calculate significant anxiety, the mean scores were calculated in both state and trait anxiety (cutoff scores for trait, >44; for state, >41 were taken) as depicted in Table 4. Mothers had significantly higher state and trait anxiety scores (mothers: 51.3/44.5; fathers: 45.5/39.9).

**Table 4:** Evaluation of total anxiety and comparison of anxiety between mothers and fathers in the study population STAI: State-Trait Anxiety Inventory; SD: standard deviation; CI: confidence interval; Df: degree of freedom

STAI SCALE	Mother mean (SD) % with significant anxiety	Father mean (SD) % with significant anxiety	95% CI of mean difference				p-value
			Lower	Upper	T	Df	
Trait	44.56 (9.6)	39.9 (8.3)	3.185	6.057	6.318	597.4	<0.001
	67.3%	45%					
State	51.30 (10.8)	45.51 (8.4)	4.252	7.330	7.390	575.43	<0.001
	76.4%	55.8%					

It was found that 67.3% of mothers had significant trait anxiety (baseline anxiety) in contrast to 45% of fathers. A total of 76.4% of mothers had state anxiety (situational anxiety) as compared to 55.8% of fathers. Also, parents with increased trait anxiety had greater state anxiety as well, since they had higher probability to worry in anxiety prone situations (p < 0.001).

## Discussion

The branch of neonatology has grown and advanced over decades. New treatment modalities and interventions have given parents of premature, extremely low-birth-weight babies and other sick infants a beacon of hope. However, with advances in the medical field as more newborns are admitted in the NICU, more parents get exposed to NICU environment and its various stressors, which has been an area of neglect since years. It is crucial for doctors and NICU staff to try to identify the stressors and to mitigate their effects. Miles in 1992 described that NICU admission of a newborn is a life-altering event for the parents. Studies done on this subject are well-documented and reviewed in Western literature; however, few Indian studies are done pertaining to parental stress and anxiety in NICU. This is the first study done in the state of Uttarakhand in Northern India. 

The current results demonstrate that parents of newborns admitted to the NICU had considerable levels of stress, which is consistent with earlier research on parental stress. The results correspond with previous studies [8-11], where moms reported greater stress levels than fathers (mothers: 9.46/fathers: 7.78, with p-value: 0.001). The four different subscales of PSS NICU scale were assessed. The highest scores in the current study for both parents were in subscale parental role alteration (mothers: 4.4/fathers: 3.33), followed by sights and sounds (mothers: 3.34/fathers: 2.79); baby looks and behaves (mothers: 1.68/fathers: 1.65), and staff communication (mothers: 0.09/fathers: 0.03). 

Maximum stress scores for both parents were obtained in subscale parental role alteration in the present study (mothers: 4.4/fathers: 3.3); similar findings were found by various researchers throughout history [9,11-13]. The first Indian study in 2012 by Chourasia, demonstrated that stress scores were highest for parental role alteration followed by looks and behavior of the baby and sights and sounds [7,8,11,12,14,15]. Not only the mothers but both parents were found to have significant stress scores in parental role alteration subscale (p < 0.001), meaning that it’s not only the mothers who are affected by the hospitalization and separation of the newborn, but paternal role is crucial for maintaining family function and stability during infants' NICU admission. It is to be known that all study on fathers has only occurred over the last one decade, before which fathers were the forgotten parent in stress evaluation in PSS NICU. 

The second scale most affected in the current study was found to be sights and sounds (mothers M: 3.3/fathers M: 2.79, p < 0.001) which caused significant stress (M > 2.9) in 68.9% of mothers and 52.2% of fathers. Followed by baby looks and behaves (mother M: 1.68/father M: 1.65), it is to be noted that mothers had higher stress scores in all PSS NICU subscales except baby looks and behaves where both the parents had equal scores (M: 1.6, similar stress levels) meaning fathers were as stressed as mothers. Similar results were noted in many studies [10,16].

In the current study, the subscale causing the least stress was staff behavior and communication; similar findings are reflected universally in all other studies [7,13,17-19]. Parental anxiety regarding the well-being, safety, and security of a child is an inevitable part of parenting. Similar to other studies, in the present study, mothers reported higher trait anxiety (baseline anxiety) scores than fathers (mother M: 44.5/ fathers M: 39.9 with p-value < 0.001). It was found that 67.3% of mothers had high trait anxiety (above cutoff level of >41) as compared to 45% of fathers [20,21]. Parents with higher trait scores were also found to have statistically significant state scores (situational anxiety) (mothers M: 51.3/fathers M: 45.5).

These results indicate that the anxiety experienced by the parents in specific anxiety prone circumstances (measured by STATE scores) is affected by the general tendency to worry (measured by TRAIT scores). Results fell in line with previous literature where high state anxiety was found in NICU parents [16].

## Conclusions

Significant levels of stress were observed in the parents of infants hospitalized in the NICU. According to the PSS NICU scale, mothers experienced higher levels of stress than fathers. Previous research has reported similar results in both Indian and Western literature. Change in parental role was the parental PSS NICU stress score subscale that was most impacted for both parents (mother: 4.4/father: 3.3 with p < 0.001). According to the STAI scale, mothers showed higher trait anxiety levels than fathers. Higher state anxiety scores (mother: 51.3/father: 45.5 with p < 0.001) were also seen in parents with higher trait anxiety ratings; substantial state anxiety was seen in 55.8% of fathers and 76.4% of mothers.
